# Aberrant structural and functional connectivity and neurodevelopmental impairment in preterm children

**DOI:** 10.1186/s11689-018-9253-x

**Published:** 2018-12-13

**Authors:** Cynthia E. Rogers, Rachel E. Lean, Muriah D. Wheelock, Christopher D. Smyser

**Affiliations:** 1 Departments of Psychiatry and Pediatrics, Washington University School of Medicine, 660 South Euclid Avenue, Campus Box 8504, St. Louis, MO 63110 USA; 2 Departments of Psychiatry, Washington University School of Medicine, 660 South Euclid Avenue, Campus Box 8504, St. Louis, MO 63110 USA; 3 Departments of Neurology, Pediatrics and Mallinckrodt Institute of Radiology, Washington University School of Medicine, 660 South Euclid Avenue, Campus Box 8111, St. Louis, MO 63110 USA

**Keywords:** Prematurity, Neurodevelopmental disorders, Functional connectivity, Structural connectivity, Magnetic resonance imaging

## Abstract

**Background:**

Despite advances in antenatal and neonatal care, preterm birth remains a leading cause of neurological disabilities in children. Infants born prematurely, particularly those delivered at the earliest gestational ages, commonly demonstrate increased rates of impairment across multiple neurodevelopmental domains. Indeed, the current literature establishes that preterm birth is a leading risk factor for cerebral palsy, is associated with executive function deficits, increases risk for impaired receptive and expressive language skills, and is linked with higher rates of co-occurring attention deficit hyperactivity disorder, anxiety, and autism spectrum disorders. These same infants also demonstrate elevated rates of aberrant cerebral structural and functional connectivity, with persistent changes evident across advanced magnetic resonance imaging modalities as early as the neonatal period. Emerging findings from cross-sectional and longitudinal investigations increasingly suggest that aberrant connectivity within key functional networks and white matter tracts may underlie the neurodevelopmental impairments common in this population.

**Main body:**

This review begins by highlighting the elevated rates of neurodevelopmental disorders across domains in this clinical population, describes the patterns of aberrant structural and functional connectivity common in prematurely-born infants and children, and then reviews the increasingly established body of literature delineating the relationship between these brain abnormalities and adverse neurodevelopmental outcomes. We also detail important, typically understudied, clinical, and social variables that may influence these relationships among preterm children, including heritability and psychosocial risks.

**Conclusion:**

Future work in this domain should continue to leverage longitudinal evaluations of preterm infants which include both neuroimaging and detailed serial neurodevelopmental assessments to further characterize relationships between imaging measures and impairment, information necessary for advancing our understanding of modifiable risk factors underlying these disorders and best practices for improving neurodevelopmental trajectories in this high-risk clinical population.

Preterm birth remains a major public health issue due to its high incidence combined with the frequency of neurodevelopmental impairments among surviving infants. In this review, we begin by highlighting the adverse effects of prematurity on trajectories across neurodevelopmental domains. Next, we discuss the increasingly established relationship between aberrant brain development and preterm birth, with particular focus on the advanced magnetic resonance imaging (MRI) techniques increasingly utilized to delineate the changes in cerebral structural and functional connectivity related to prematurity. We then review selected studies from the extant literature which suggest that prematurity-associated changes in cerebral structural and functional connectivity may underlie the neurodevelopmental impairments common among prematurely born children and adults. Finally, we conclude by detailing relevant clinical and social variables that may influence these relationships in this high-risk clinical population.

## Prematurity and neurodevelopmental disorders

Premature birth affects more than 500,000 newborns in the USA each year, occurring in approximately 10% of all births in 2016 [1]. Survival rates for these infants have improved dramatically due to advances in perinatal and neonatal care. In contrast to this improvement in mortality, long-term neurodevelopmental outcomes have not improved, with preterm birth remaining a leading cause of neurological disabilities in children [2]. These surviving preterm children face a range of neurodevelopmental and neurobehavioral challenges [3–7], with more than 30% experiencing impairments across multiple neurodevelopmental domains [8]. Children delivered very preterm (VPT; born at ≤ 32 weeks’ gestation) typically face disproportionate risk, with infants born earliest facing the highest rates of developmental disability [9]. However, these adverse effects are not universal, with widely varied outcomes among preterm children with similar neonatal clinical phenotypes. Critically, the associated costs in caring for these children are enormous, amounting to more than $25 billion annually in the USA alone [10].

Among preterm children, prominent neurodevelopmental difficulties are seen across motor, cognitive, language, and social-emotional domains [11–14]. These areas warrant particular focus due not only to their critical functional importance, but also to their significant impact on quality of life, including poor peer relationships [15] and academic underachievement [16–18]. Over 50% of children diagnosed with cerebral palsy are born preterm, with the greatest likelihood among those born at the earliest gestational ages [19]. An even larger proportion of preterm children experience other more subtle fine and gross motor problems, with approximately 40% displaying mild to moderate motor impairment [12]. Similarly, 15–20% of intellectual disabilities and 10–15% of other learning disorders are attributable to preterm birth. VPT children obtain Full Scale Intelligence Quotient (IQ) scores up to 10 points lower than term children [20, 21]. Furthermore, VPT children consistently perform worse than term-born peers on executive function tasks assessing planning, fluency, working memory, and response inhibition [22–24]. Preterm children also demonstrate problems in selective, sustained, and executive attention, with up to 41% of VPT and 62% of extremely preterm (born at < 28 weeks’ gestation) children in the impaired range [25–28]. Further, large effect sizes have been reported for executive shifting and divided attention [25, 26, 29], suggesting VPT children particularly struggle with top-down control of attention processes. In addition, approximately 35% of children born between 31 and 34 weeks’ gestation demonstrate language impairments at preschool-age, with rates as high as 48% for children born at less than 30 weeks gestation [30]. Deficits in both receptive and expressive language domains persist into school age, affecting skills such as word finding, perception, grammar, dialog, and linguistics [30–34]. Critically, across each of these neurodevelopmental domains, preterm birth remains a strong risk factor for impairment even after accounting for sociodemographic risk [19, 35].

More recently, elevated rates of social-emotional deficits and psychiatric disorders have been recognized among children born preterm, with increasing numbers of reports detailing the “preterm behavioral phenotype” [36], comprised of inattention, anxiety, and social-communication deficits [37]. These comorbid symptoms and the related disorders of Attention-deficit hyperactivity disorder (ADHD), anxiety, and autism spectrum disorder (ASD) are two to four times more common among preterm children [5, 38–43]. As with other neurodevelopmental impairments, children born VPT are at greatest risk for these social-emotional impairments and psychiatric diagnoses [36]. Further, studies examining the trajectory of these symptoms demonstrate their persistence into adolescence [5, 44–49]. Importantly, rates of these disorders remain elevated even after accounting for the increased frequency of other neurodevelopmental disabilities, including motor and intellectual impairments [36].

## Assessment of functional and structural connectivity in preterm children using MRI

The timing of key interrelated neurobiological processes underlying development of early cerebral functional and structural connectivity make the preterm brain uniquely vulnerable to the perturbations that have been associated with common neurodevelopmental disorders. Most notably, this includes processes such as neuronal migration, synaptogenesis, cortical folding, emergence of thalamo-cortical connections, and myelination [50]. Early investigations of children born preterm employed conventional MRI to characterize the alterations in cerebral structural development associated with preterm birth [51–55]. These predominantly cross-sectional investigations focused on metrics of brain growth, regional brain volumes, and cortical folding, demonstrating atypical patterns of maturation throughout the brain across techniques in preterm children [56–58]. However, these modalities provided only limited ability to elucidate the alterations in cerebral development that lead to neurodevelopmental deficits; information critical for understanding the pathway to disability.

Advanced MRI techniques, including resting state-functional MRI (rs-fMRI) and diffusion MRI (dMRI), provide powerful, non-invasive tools with high sensitivity for delineating alterations in the developing brain. rs-fMRI is used to detect temporal correlations in spontaneous, low-frequency fluctuations in blood oxygen level-dependent signal, thereby identifying functional connectivity networks from data acquired without requiring subjects to perform tasks during acquisition [59–61]. These resting state networks incorporate gray matter regions known to be anatomically connected and co-activated by task performance [60, 62, 63]. dMRI characterizes cerebral structural connectivity through quantification of water displacement within the white matter microstructural architecture [64–66]. In many ways, these modalities are well-suited for investigations of infants and pediatric populations; from a study lasting minutes in duration on an infant at rest, robust measures of global functional and structural connectivity can be obtained. Further, both modalities have been used successfully to investigate cerebral connectivity in VPT adults and older pediatric populations, demonstrating atypical connectivity patterns which correlate with neurodevelopmental disability [57, 67–71].

Unique methodological challenges have now been overcome to successfully study neonates and young children using rs-fMRI and dMRI, including scan sequence specification, scanning of non-sedated subjects, effects of small brain sizes on atlas registration, and development of data processing streams [72–75]. Our group and others have subsequently used these techniques to identify immature forms of multiple canonical resting state networks and white matter tracts throughout the brain as early as 26 weeks postmenstrual age (PMA). These systems reflect the functional and structural topography of the developing brain, gradually maturing with advancing age [74]. Our recent applications of rs-fMRI and dMRI demonstrate infants possess a functional and structural network architecture similar to that described in adults, with maturation rates emulating known histological evidence regarding brain development [74, 76, 77]. For example, networks (e.g., somatomotor, auditory, visual networks) and tracts (e.g., corticospinal tracts, optic radiations) in areas of the brain known to develop early demonstrate mature topology by term equivalent PMA. In contrast, networks (e.g., default mode [DMN], frontoparietal [FPN], cingulo-opercular [CO] networks), and tracts (e.g., cingulum bundle, uncinate) located in higher-order association cortices involved in top-down control of emotion regulation, attention, and cognition do not demonstrate adult-like topology until later in life.

**Fig. 1:**
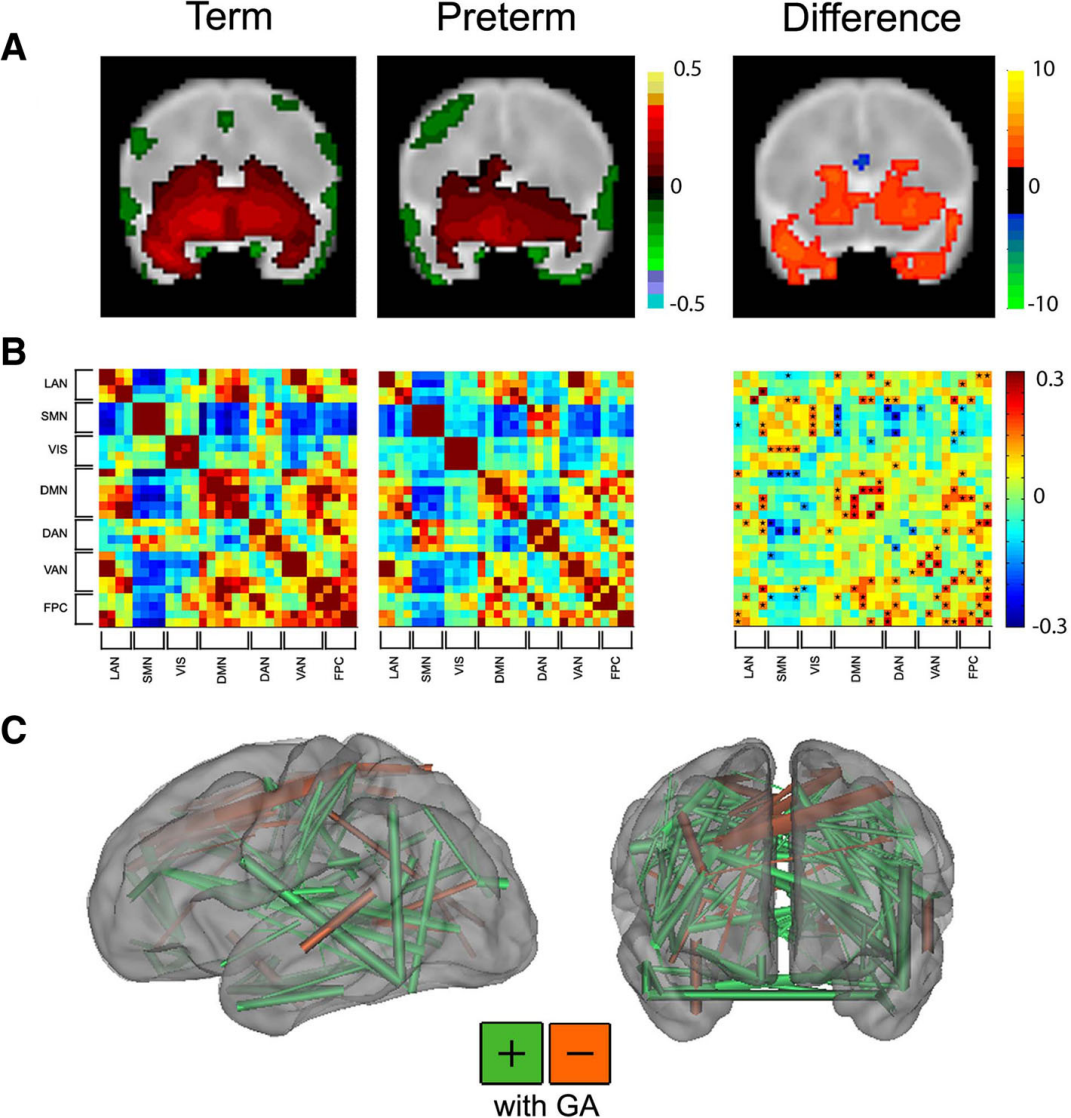
Functional connectivity differences between term and very preterm infants. **a** Left: group mean amygdala resting state-functional connectivity correlation maps for full-term and very preterm infants scanned at term equivalent postmenstrual age; right: *z* scores demonstrating group differences in connectivity obtained from voxelwise *t* test. Blue voxels denote areas with greater negative correlations and orange voxels denote areas with greater positive correlations in term infants. Results thresholded using |*z*| > 2.25 and 53 contiguous voxels achieving whole-brain false-positive rate of 0.05. Adapted with permission from Rogers CE, et al. JAACAP. 2017; 56(2):157-166. **b** Left: group mean covariance matrices representing multiple canonical RSNs for full-term and very preterm infants at term equivalent postmenstrual age; right: difference between these two results (term minus preterm). Black stars denote cells with between group difference on two-tailed Mann-Whitney *U* test (*p* < 0.05; multiple comparisons uncorrected). Adapted with permission from Smyser CD, et al. Cerebral Cortex. 2016; 26(1):322-333. **c** Functional connections important for differentiating full-term versus very preterm infants using support vector machine-multivariate pattern analysis to analyze data from 244 regions of interest located throughout the brain. Connections stronger in term infants are shown in green; those stronger in very preterm infants are in orange. The caliber of each connection is weighted by the difference magnitude. Adapted with permission from Smyser CD, et al. NeuroImage. 2016; 136:1-9

Further, these methods are sensitive to the changes in functional and structural connectivity associated with premature birth (Fig. 1). Across rs-fMRI investigations, infants born prematurely demonstrate similar overall resting state network topography to term-born infants scanned at comparable PMA, though with weaker intrinsic brain activity. The magnitude of these differences in network amplitude and dimensionality differ by network and are typically most prominent in those located in higher-order association cortices [74, 77, 78]. Infants with forms of white matter injury common in preterm populations (e.g., intraventricular hemorrhage, cystic periventricular leukomalacia) demonstrate aberrant network development, dependent upon severity and proximity to the injury site [79]. Interrelated investigations of structural connectivity using dMRI also demonstrate comparable regionally specific differences in gray and white matter microstructural development between preterm and term-born infants [80–86]. Across these studies, prematurely born infants demonstrate delayed white matter tract development, with susceptibility to specific clinical factors (e.g., antenatal steroids, white matter injury) also reported. Further, these neuroimaging data are conducive to technically sophisticated analysis approaches designed to investigate complex patterns in neuroimaging data, such as graph theory and machine learning [76, 87–91]. Use of these methods in neonates and older pediatric populations have demonstrated the importance of connectivity within and between networks for differentiation of term- and prematurely born infants and continuous measure (i.e., birth gestational age) prediction [92–94]. These studies provide converging lines of evidence suggesting neurodevelopmental impairment may directly correlate with disruptions in specific structural and functional systems.

## Prematurity-related changes in functional and structural connectivity and developmental impairment

There is a small, but burgeoning literature investigating the relationship between cerebral functional and structural connectivity changes and motor, cognitive, language, and social-emotional outcomes in prematurely born children [54, 58, 81, 95–103]. For brevity, across each of these domains, we highlight representative studies which have served to increasingly identify the links between measures of aberrant brain connectivity and adverse neurodevelopmental outcomes, beginning during the neonatal period and extending into adulthood. As the methods for application of the modality were established earlier, many of these studies utilized dMRI, though an increasing number of recent studies also include rs-fMRI. Further, many of these investigations have been cross-sectional and focused on older populations, though longitudinal investigations including neonatal data are now being published.

### Motor

**Fig. 2:**
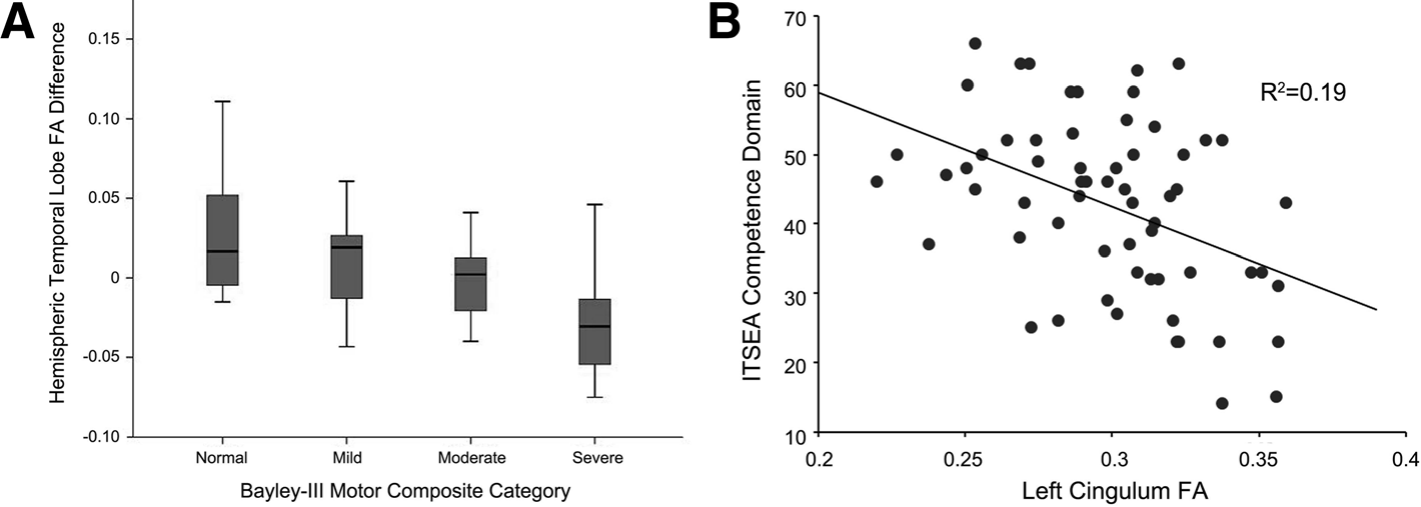
Relationship between regional neonatal structural connectivity measures and developmental outcomes in preterm children. **a** Boxplots of hemispheric asymmetry between neonatal left and right inferior temporal lobe white matter fractional anisotropy in very preterm infants scanned at term-equivalent age and Bayley-III Motor Composite Categories based upon assessments performed at age 2 years, corrected. **b** Regression plot demonstrating the relationship between fractional anisotropy in the left cingulum bundle at term-equivalent age and competence scores on the Infant Toddler Social Emotional Assessment (ITSEA) tool at age 2 years, corrected. Note the association between more impaired (lower) ITSEA competence scores and higher FA (*p* = .001). Adapted with permission from Rogers CE, et al. Pediatric Research. 2016; 79(1-1):87–95

dMRI and rs-fMRI have been increasingly used to demonstrate clinically relevant alterations in key white matter tracts and the motor network in prematurely born infants and children. Recently, higher mean and radial diffusivity within the splenium of the corpus callosum and lower fractional anisotropy (FA) in the left inferior temporal lobe in VPT infants, indicating delayed and/or aberrant tract development, were associated with worse motor functioning at age 2 years (Fig. 2) [81, 99]. Similar longitudinal relationships persist into later childhood, as VPT infants with decreased neonatal FA in inferior occipital and cerebellar regions demonstrated greater motor impairments at age 7 years [100]. Further, at age 7, VPT children with a higher degree of motor impairment demonstrated reduced structural connectivity within the precuneus, inferior parietal cortex, and temporal lobes in a network-based analysis [101]. Comparable patterns are present in adulthood, with preterm-born adults found to have lower FA in the corpus callosum, inferior longitudinal fasciculus, inferior fronto-occipital fasciculus, and external capsule demonstrating worse visual-motor integration and motor abilities [104]. White matter injury affects these brain-behavior relationships, with motor-impaired VPT children with moderate-severe white matter injury demonstrating lower corpus callosum FA than VPT children with normal motor outcomes at age 7 [105]. Further, children with periventricular leukomalacia and gross motor impairment demonstrated reduced corticospinal tract size [106] and decreased FA within the corticospinal tract and cerebellar peduncles [107]. Similar findings have been reported using rs-fMRI, with investigations of prematurely born children, adolescents, and adults with spastic diplegic cerebral palsy due to periventricular leukomalacia demonstrating aberrant motor network connectivity in relation to term-born peers that correlated with severity of motor impairment [108, 109].

### Cognitive

Alterations in cerebral white matter microstructure identified using dMRI have also been linked to adverse cognitive outcomes in preterm children [101, 110–112]. A recent prospective longitudinal study including serial dMRI scans in preterm infants at birth, term equivalent PMA and 2 and 4 years demonstrated that slower rates of change in mean diffusivity (MD) of the internal and external capsules from birth to age 4, also reflecting delayed and/or aberrant tract development, were associated with poorer intellectual ability at age 4 [112]. In addition to these neonatal findings, reduced FA in the uncinate fasciculus, corticospinal tract, cingulum bundle, inferior frontal fasciculus, inferior frontal-occipital fasciculus, superior longitudinal fasciculus, and anterior thalamic radiations has also been associated with worse intellectual and/or executive function skills in preterm children and adolescents [110, 111]. Further, VPT children with cognitive impairment demonstrate reduced connections in a white matter network including the thalamus, hippocampus, paracentral lobule, posterior cingulate, parietal and occipital cortices, and frontal and temporal gyri compared to non-impaired preterm children in a network-based analysis of white matter structural connectivity graphs [101].

### Language

Multiple studies have also linked aberrant structural and functional cerebral development with poor language outcomes in preterm children. A serial MRI study reported that greater increases in axonal diffusivity of the left posterior thalamic radiation from term-equivalent PMA to age 4 years was associated with poorer receptive and expressive language ability at age 4 [112]. Higher MD in the centrum semiovale and left superior temporal gyrus has also been linked to poorer language outcomes in preterm children [113, 114]. Consistent with these early childhood findings, alterations in the uncinate fasciculus, splenium of the corpus callosum, and anterior commissure explained up to 57% of variability in language outcomes among preterm adolescents [115]. Recent rs-fMRI investigations have also shown that preterm children and adolescents demonstrate persisting alterations in language networks compared to term-born peers [116]. Specifically, preterm children demonstrate increased connectivity strength between the language network and other regions throughout the brain, with decreased right hemisphere lateralization [117, 118]. These differences have been related to language performance, with preterm adolescents demonstrating weaker bilateral connectivity between left and right superior temporal regions also demonstrating poorer language ability at age 14–15 years [115, 116], with other regionally specific relationships also reported [68, 119, 120].

### Social-emotional

**Fig. 3:**
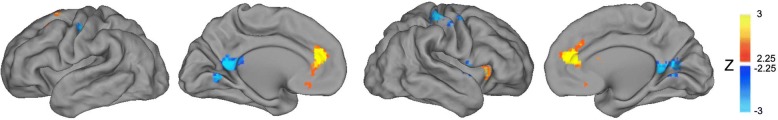
Relationship between neonatal amygdala functional connectivity and social-emotional outcomes in preterm children. Results from whole-brain analysis investigating the relationship between neonatal functional connectivity of left amygdala and internalizing scores on the Infant Toddler Social Emotional Assessment (ITSEA) tool at age 2 years, corrected. Images demonstrate higher total internalizing domain scores were positively correlated with functional connectivity measures between the left amygdala and the medial prefrontal cortex, right anterior insula, and superior frontal cortex. Results thresholded using |*z*| > 2.25 and 53 contiguous voxels achieving whole-brain false-positive rate of 0.05. Adapted with permission from Rogers CE, et al. JAACAP. 2017; 56(2):157–166

Symptoms of ADHD, anxiety, and ASD comprising the preterm behavioral phenotype have also been linked to altered neonatal structural and functional connectivity in key brain regions [96, 121–142]. Recent evidence suggests that preterm birth may predispose children to higher rates of emotion dysregulation and social-emotional disorders due to stress experienced during the NICU hospitalization via changes in hypothalamic-pituitary-adrenal axis function [143–146] and brain connectivity [73, 147]. For instance, alterations in connectivity of the glucocorticoid-rich amygdala [148], which has a prominent role in emotion processing [149–151], have been linked to NICU stress exposure in preterm infants [147]. It has also been shown that neonatal rs-fMRI measures between the amygdala and regions of key cortical networks, including the DMN, FPN, and CO, are related to variability in anxiety symptoms in VPT infants at 2 years (Fig. 3) [152, 153]. Aberrant dMRI measures of white matter tracts related to ADHD, anxiety, and ASD symptoms, such as frontostriatal circuits and frontolimibic regions including the cingulum and uncinate [96, 131–135], have also been associated with these same symptom domains in VPT children [81, 98, 154].

Overall, these lines of converging evidence relating functional and structural connectivity to neurodevelopmental outcomes in preterm children indicate that for early developing white matter tracts and functional networks there are typically well-defined, regionally specific relationships between aberrant connectivity and domain-specific neurodevelopmental impairment. In contrast, abnormalities in tracts connecting key regions within functional networks such as the DMN, FPN, and CO, including the corpus callosum, uncinate, and cingulum, have been linked to impairment across multiple domains. In combination, these results suggest that alterations in structural connectivity underlie the abnormal functional connectivity patterns identified in preterm children, though in a tract- and network-specific manner, and that these differences play a critical role in the increased rates of adverse outcomes in this high-risk clinical population. Further, this work highlights our evolving understanding of the interrelationship between early structural and functional connectivity and the deleterious effects of preterm birth on brain development and neurodevelopmental outcomes.

## Clinical variables linked to developmental impairment in preterm children

While the highlighted research suggests that prematurity-associated alterations in structural and functional connectivity underlie neurodevelopmental impairments in preterm children, other clinical and social factors likely modify this risk. Two important considerations include sociodemographic risk factors and heritability. Preterm children experience higher rates of sociodemographic risk factors known to be associated with developmental deficits, with preterm birth disproportionately occurring among mothers from socially disadvantaged backgrounds [155, 156]. The odds of VPT delivery are 1.03–1.27 times higher in mothers living below the poverty threshold [157–159], with these mothers typically having low levels of education and high levels of assistance from public health care programs [156, 160, 161]. Among VPT children, poverty is a particularly strong predictor of cognitive, motor, and language outcomes [162–171]. Other psychosocial risk factors more common among preterm infants, including maternal depression [172, 173], high parenting stress [174, 175], and unsupportive maternal-child interactions [176–178], have also been linked to adverse psychiatric outcomes [179–187]. For example, we reported maternal depression during early childhood-mediated risk for anxiety disorders associated with preterm birth [188]. In addition, these same risk factors have been linked to changes in brain development, with exposure to poverty and unsupportive caregiving impacting functional and structural brain development in offspring [186, 189–191]. Thus, preterm birth both increases the likelihood of experiencing early psychosocial adversity and alters functional and structural development of the neonatal brain. Further, the developing brain may remain highly vulnerable to continued alterations from repeated exposures to psychosocial adversity extending beyond the neonatal period.

Another key and understudied risk factor among preterm children is heritability. Studies investigating heritability suggest that family background determines the lower and upper limits of the range in which a heritable and continuously distributed trait may be expressed, but that neurodevelopmental disorders increase the phenotypic variability of trait expression during childhood [192, 193]. For instance, maternal intellectual ability has a direct influence on her children’s intellectual development because it is a genetically based and heritable trait [194]. Preterm children born to mothers with low levels of intellectual ability may therefore be at higher risk of poor outcomes. Indeed, our analysis of maternal intellectual ability demonstrated that maternal IQ scores were associated with both preterm and term child IQ and language scores at age 5 years [195]. However, the association between maternal IQ and child IQ and language outcomes was weaker for preterm children, indicating preterm birth itself was an important factor explaining intellectual and language development. Further, heritability is an important variable for social-emotional development and psychiatric symptoms underlying the preterm behavioral phenotype, as ADHD, ASD, and anxiety symptoms are all highly heritable [196–199]. In some cases, the heritability of social-emotional symptoms may confound the relationship between prematurity and social-emotional development. For instance, substance-abusing mothers are more likely to both have ADHD [200, 201] and anxiety [202] and to deliver preterm [203, 204]. A similar relationship could exist between the highly related variables of maternal depression and both preterm delivery [205] and childhood anxiety [206]. These findings highlight the need to assess psychosocial risk factors and heritability among families in all research investigating links between preterm birth and neurodevelopmental outcomes.

## Future directions and conclusions

Continued research remains necessary to both further delineate the relationships between imaging measures and neurodevelopmental impairment in prematurely born children and better characterize the role of modifiable risk factors such as psychosocial adversity in this trajectory. While MRI affords several advantages for studying these associations, including improved spatial resolution and anatomic specificity, future investigations may utilize other complementary modalities for assessing brain development and function. These include functional near infrared spectroscopy (fNIRS), which measures hemodynamic contrasts [207–209] and electroencephalography (EEG), which assesses the coherence of cortical electrical activity and has been used to successfully model brain connectivity-behavior associations [210]. In addition, diffuse optical tomography (DOT) enables measurements of functional connectivity which align with rs-fMRI, though with a more limited field of view [211, 212]. Limitations notwithstanding, these portable methods can be readily employed to perform serial studies at the bedside, providing avenues for novel investigation by enabling the study of clinical populations of interest unable to undergo MRI.

Future work should also focus on extending longitudinal evaluations of preterm children across early childhood, leveraging recent advances in MRI acquisition and analysis methods and incorporating advances developed and implemented among other clinical populations. For example, the Infant Brain Imaging Study has performed longitudinal MRI scanning of infants at risk for autism beginning at 6 months of age with repeat MRI scans at 12 and 24 months, reporting changes in both structural and functional connectivity parameters utilizing longitudinal analyses of brain development and innovative brain-behavior analyses [127, 213]. More recently, the UNC/UMN Baby Connectome Project (BCP), building on sequence development from the Human Connectome Project, is studying longitudinal brain development across the first 5 years of life, including imaging preschool-age children in an awake state [214]. The BCP aims to provide innovative data regarding early typical structural and functional brain development through improved acquisition resolution, optimized diffusion sequences, and frequent longitudinal sampling across early childhood. While substantive technical challenges remain, including best practices for studying children in the setting of evolving tissue contrast and registration of individual imaging data sets across multiple time points, these methods are being increasingly established and can be employed at most institutions.

Collectively, the studies reviewed here and elsewhere [215] provide converging evidence suggesting neurodevelopmental disabilities common in prematurely born children directly relate to early disruptions and/or remodeling of specific functional and structural networks [102]. Continued use of advanced neuroimaging techniques in combination with detailed serial neurodevelopmental assessments as part of longitudinal studies of preterm brain development has great potential to advance the field of developmental neuroimaging. Critically, these studies will provide improved understanding of the aberrant trajectories of structural and functional connectivity in prematurely born children and the role of these differences in adverse outcomes. Further, these investigations will provide valuable insights into how psychosocial and familial factors impact not only neonatal brain development, but also the nature and evolution of subsequent alterations during early childhood. Ultimately, this information will prove valuable for both advancing our understanding of modifiable factors underlying these disorders and defining best practices for improving neurodevelopmental trajectories in this high-risk population.

## Abbreviations

ADHD: Attention-deficit hyperactivity disorder
ASD: Autism spectrum disorder
CO: Cingulo-opercular network
DMN: Default mode network
dMRI: Diffusion magnetic resonance imaging
FA: Fractional anisotropy
FPN: Frontoparietal network
IQ: Intelligence quotient
MD: Mean diffusivity
MRI: Magnetic resonance imaging
PMA: Postmenstrual age
rs-fMRI: Resting state-functional magnetic resonance imaging
VPT: Very preterm
